# ProMMF_Kron: a multimodal deep learning model for immunotherapy response prediction in stomach adenocarcinoma

**DOI:** 10.3389/fimmu.2026.1602846

**Published:** 2026-02-10

**Authors:** Chenchen Wang, Weikun Liu, Dongmei Ai, Xiuqin Liu

**Affiliations:** School of Mathematics and Physics, University of Science and Technology Beijing, Beijing, China

**Keywords:** microsatellite instability, ICI therapy, multimodal data, deep learning, stomach adenocarcinoma

## Abstract

**Background:**

Immune checkpoint inhibitor (ICI) therapy has significantly improved treatment outcomes for various cancers by enhancing T cell-mediated anti-tumor immune responses. However, accurately predicting patient response to ICI treatment remains a major challenge due to the risk of immune-related adverse events. Microsatellite instability (MSI), as an important molecular biomarker characterized by high mutation rates and abundant tumor neoantigen production, has been demonstrated to effectively predict clinical benefits from immunotherapy. In gastric adenocarcinoma (STAD) patients, approximately 22% exhibit the MSI subtype while the majority are microsatellite stable (MSS). This significant molecular heterogeneity underscores the urgent need to develop reliable predictive tools.

**Methods:**

To address this problem, we developed a multimodal deep learning model named ProMMF_Kron based on a multicenter dataset comprising 282 patients. The model employs a two stage feature fusion strategy: first extracting key features from both molecular profiles and pathological images through differential gene analysis and a pretrained deep convolutional neural network, respectively; then designing a sophisticated fusion architecture incorporating Kronecker product operations and back-projection modules to achieve efficient interaction between gene expression features and pathological image features. The dataset was partitioned into training, validation, and testing sets at a ratio of 6:2:2.

**Results:**

Experimental results demonstrate that the ProMMF_Kron model effectively distinguishes between MSI and MSS subtypes (MSI versus MSS) and exhibits competitive predictive performance on independent test datasets, achieving an AUC of 0.96 (95% CI: 0.89-1.00), outperforming traditional single-modality prediction models (3.2% AUC improvement) and other multimodal fusion approaches (4.3% AUC improvement). Further validation confirms the model’s excellent stability and generalization capability, maintaining high predictive accuracy on colorectal cancer (CRC) dataset.

**Discussion:**

Through bioinformatics analysis and feature visualization techniques, this study also reveals potential associations between key molecular biomarkers and critical immune regulatory pathways, providing a powerful decision-support tool for precision immunotherapy in gastric cancer with substantial clinical translation value and application prospects.

## 1 Introduction

Immune checkpoint inhibition (ICI) therapy enhances anti-tumor immune responses by blocking inhibitory signals that are activated on T cells (1). Immune checkpoints are primarily inhibitory receptors on T cells that prevent the immune system from mistakenly attacking normal tissues, thus causing autoimmunity, by regulating T cell activation and function (2–4). However, tumor cells exploit this mechanism by causing their surface ligands to bind to inhibitory receptors on T cells, sending inhibitory signals that suppress T cell anti-tumor responses and allowing the tumor to evade the immune system (5, 6). By blocking these interactions, immune checkpoint inhibitors release T cells from inhibition, enabling them to continue attacking tumor cells (7–9).

Currently, two types of immune checkpoint inhibitors have been approved for the treatment of various cancers with remarkable clinical efficacy: cytotoxic T-lymphocyte-associated antigen 4 (CTLA-4) antibodies, and programmed death receptor 1 (PD-1) and its ligand PD-L1 antibodies (10–14). CTLA-4 antibodies were the first class of immunotherapeutic drugs approved by the U.S. Food and Drug Administration (FDA) (15). Clinical trials have demonstrated that the anti-CTLA-4 antibody ipilimumab significantly prolongs overall survival in patients with metastatic melanoma (16, 17). Recent studies have shown that the fully human IgG4 PD-1 immune checkpoint inhibitor nivolumab, by blocking the PD-1-mediated signaling pathway, provides superior survival benefits compared to the traditional chemotherapy drug docetaxel in patients with advanced non-squamous non-small cell lung cancer (18, 19). Notably, the latest research indicates that a dynamic attention multimodal model (DyAM) integrating radiomics (CT imaging), pathomics (PD-L1 immunohistochemistry), and genomic features can significantly enhance the predictive efficacy of PD-(L)1 immunotherapy response in non-small cell lung cancer patients, highlighting the value of cross-modal feature integration in precision immunotherapy (20). Recent multimodal deep learning studies have demonstrated that integrating imaging, pathological, and clinical data can significantly improve the prediction of immunotherapy response and survival outcomes (21–23).

In addition, recent advances in deep learning have also been successfully applied in diverse domains, such as image segmentation (24, 25) and healthcare workflow optimization (26), providing methodological insights relevant to our study.

Although these advances underscore the potential of ICIs, their broader application has been accompanied by immune-related toxicities, including neurological complications such as encephalitis, myelitis, aseptic meningitis, peripheral neuropathy, myasthenia gravis, and myositis, which have become major challenges for many patients (27).

To optimize the application of immunotherapy and reduce adverse effects, reliable indicators are needed to evaluate patients’ suitability for ICI therapy. Several predictive biomarkers have been identified to help determine the likelihood of patient benefit, among which microsatellite instability (MSI) is a key indicator. Microsatellites are short tandem repeat sequences in DNA that undergo alterations when the DNA mismatch repair (MMR) system is defective, leading to MSI (28). MSI tumors typically exhibit high mutational burden and generate more neoantigens, making them more susceptible to immune system recognition and attack. Consequently, MSI-positive patients generally show greater sensitivity to ICI therapy (29–31). However, laboratory tests (such as PCR or immunohistochemistry) for determining microsatellite status are associated with operational complexity, long turnaround times, and high costs, which limit their widespread use in clinical practice.

According to the 2020 global cancer statistics, gastric adenocarcinoma ranks as the fifth most common cancer worldwide and the fourth leading cause of cancer-related mortality (32). Approximately 22% of gastric adenocarcinoma (STAD) cases exhibit microsatellite instability (MSI), while the remaining are microsatellite stable (MSS) type (33). This classification holds significant clinical implications, as MSI tumors with their high mutational burden tend to respond better to immune checkpoint inhibitor (ICI) therapy, whereas MSS patients generally show poor response to ICIs. This underscores the urgent need to develop alternative treatment strategies specifically for MSS patients.

Currently, researchers have developed various machine learning models to predict MSI status for accurately identifying patients who may benefit from ICI therapy. However, significant limitations remain: On the one hand, most studies rely solely on unimodal data—for example, Kather et al. (34) used ResNet18 to predict MSI status based on TCGA gastrointestinal tumor histopathology images, and Wang et al. (35) constructed an SVM prediction model using whole-exome sequencing (WES) mutation data. On the other hand, existing research has primarily focused on colorectal cancer (CRC), such as the multimodal deep learning framework proposed by Qiu et al. (36), which innovatively integrates H&E-stained histopathology images with multi-omics data; the decision tree model employed by Bodalal et al. (37); and the Bayesian fusion model developed by Liu et al. (38). Although recent studies suggest that integrating histopathological and molecular data through deep learning techniques can improve MSI prediction accuracy (36–38), these advances remain underutilized in gastric adenocarcinoma research.

In this study, we propose progressive fusion (Pro-Fusion) with Kronecker product (ProMMF_Kron), a novel deep learning framework that integrates gene expression profiles and histopathological images for MSI status prediction in STAD. Our framework introduces two key technical innovations: (1) An iterative deep fusion module enabling dynamic refinement of cross-modal features through multi-step back-projection, which facilitates fine-grained interactions between pathological and genomic data to enhance both classification performance and feature representation richness: (2) A flexible architecture specifically designed to accommodate the characteristics of both histopathology and genomics, endowing the model with potential for cross-cancer generalization. This multimodal approach effectively captures complementary MSI features that are inevitably missed by single-modality methods, demonstrating superior predictive accuracy compared to existing approaches. Importantly, our system synergistically analyzes genomic data (including gene expression profiles and genetic variants) with pathological images (containing morphological and tissue architecture information), providing comprehensive tumor characterization at both molecular and histological levels. This breakthrough not only addresses the technical challenges in STAD MSI prediction, but also establishes a new paradigm for identifying patients who may benefit from immunotherapy.

## 2 Materials and methods

### 2.1 Data materials

The pathology image data used in this study comes from the pre-processed block-level dataset provided by Kather et al. (34). This dataset, derived from TCGA-STAD (The Cancer Genome Atlas Stomach Adenocarcinoma) cohort, includes 216,811 block-level images from 315 STAD patients. The images underwent a series of preprocessing steps to ensure data consistency and high quality, which primarily included automatic tumor region detection, tessellation of the tumor regions into 256 μm × 256 μm tiles, and color normalization using the Macenko method. The color normalization is a critical step aimed to minimize inter-slide staining variations caused by differences in staining protocols or scanners, thereby ensuring that the deep learning models focus on biologically relevant morphological features rather than technical artifacts (34). Additionally, we obtained the corresponding RNA-Seq count data for these patients from the Cancer Genome Atlas (https://cancergenome.nih.gov). However, gene expression data were not available for all 315 patients; after matching the pathological images with the available genomic profiles, only 282 patients were included in the final analysis. The detailed clinico-pathological characteristics of all patient cohorts are presented in **Supplementary Table S1** and the patient ID in **Supplementary Table S2**.

Based on these data characteristics, we established a rigorous patient-level data partitioning protocol: First, we ensured that all pathological images and their corresponding gene expression data from the same patient were consistently assigned to the same dataset. Second, we divide the 282 patients (rather than individual image tiles) into training, validation, and test sets in a 6:2:2 ratio. During the partitioning process, we fixed the random seed (random_state = 2024) to ensure the reproducibility of our experiments. This partitioning protocol effectively prevents potential information leakage that might occur when samples from the same patient are dispersed across different datasets by strictly maintaining patient-level data integrity. RNA-Seq count data were converted to TPM, log2(TPM + 1)-transformed, and standardized by feature-wise z-score normalization. As TCGA Level 3 data are already processed and normalized, no additional batch-effect correction was applied.

To assess the model’s cross-cancer applicability, we obtained 360 formalin-fixed paraffin-embedded (FFPE) colorectal cancer (CRC) samples from the TCGA-CRC-DX cohort, among which 357 cases (**Supplementary Table S2**) had matched gene expression data. The data sources and preprocessing pipeline were consistent with those used for the TCGA-STAD gastric cancer dataset, all originating from The Cancer Genome Atlas (https://cancergenome.nih.gov). During data partitioning, we strictly adhered to patient-level separation principles to ensure all pathological images and corresponding gene expression data from each patient were allocated to the same dataset. The 357 cases were randomly divided into training, validation, and test sets in a 6:2:2 ratio, with a fixed random seed (random_state = 2024) to ensure experimental reproducibility.

### 2.2 Feature extraction

#### 2.2.1 Feature extraction of gene expression data

The R package “DESeq2” was used to identify differentially expressed genes in the RNA-Seq data from the training set. Patients were categorized into MSI and MSS groups, with genes exhibiting a logFC > 2 and a p-value < 0.05 labeled as up-regulated, and genes with logFC < -2 and p-value < 0.05 labeled as down-regulated. Volcano plots were generated using the “ggplot2” package to visualize gene expression differences. The DESeq2 analysis aimed to identify candidate features for deep learning modeling, rather than definitive gene sets for biological interpretation. Therefore, we used nominal p-values with fold-change thresholds instead of multiple comparison correction to retain potentially informative features. After screening for differential genes, we performed Gene Ontology (GO) analysis and Kyoto Encyclopedia of Genes and Genomes (KEGG) analysis using the R package “ClusterProfiler”. The aim of these analyses was to better understand the roles of these differential genes in immune-related pathways and to assess their potential functions in immune responses. Through GO analysis, we identified the biological processes, cellular components, and molecular functions associated with these genes, revealing their involvement in key immune processes such as immune response, cell signaling, and inflammation. KEGG analysis, on the other hand, provided insights into the participation of these genes in known immune pathways, helping us further understand their roles within the immune microenvironment and potential immune regulatory mechanisms.

#### 2.2.2 Feature extraction of pathology image data

In this study, we use a pre-trained ResNet50 model for feature extraction from patient tissue slice images. ResNet50 is a deep convolutional neural network with 50 layers, pre-trained on the large ImageNet dataset, and is equipped with robust feature learning capabilities. Through pre-training, the model learns to capture generic image features such as edges, textures, and shapes, allowing it to quickly adapt and efficiently extract relevant features for new tasks.

Specifically, the slice images of each patient are input into the pre-trained ResNet50 model. The model processes the images layer by layer through forward propagation, involving operations such as convolution, normalization, ReLU activation, and pooling layers. As the network deepens, the raw high-dimensional image data is transformed into a multi-layered feature map. In the global average pooling layer, the model compresses the feature representation of each image into a 2048-dimensional vector, which retains critical information while significantly reducing dimensionality, enhancing the efficiency of subsequent analysis. These feature vectors are then passed into an attention-based deep multi-instance learning (AMIL) model, as described in Section 2.3.1, which integrates the image features from each patient, ultimately enabling the classification and prediction of the patient’s MSI versus MSS status.

### 2.3 Pathology image aggregation and multimodal fusion at patient level

#### 2.3.1 Attention-based deep multi-instance learning

Let *M* represent the total number of slices in the dataset of all patients, where $M=\sum_{i=1}^{n}M_{i}$, and $M_{i}\;$ is the number of slices for the *i*-th patient. Using ResNet50, the feature representations of all $M_{i}$ slice images for each patient are transformed into 2048-dimensional vectors. These feature vectors are then mapped through a linear layer into a 512-dimensional vector ${\text{h}}_{m}\in {\mathbb{R}}^{512}$. Subsequently, the attention score $a_{m}$ for each image is calculated (39), with ${\text{U}}_{a}$, ${\text{V}}_{a}{\;\text{and}\;\text{W}}_{a}$ representing the weight parameters.


$$a_{m}=\frac{\text{exp}\{W_{a}(\text{tanh}(V_{a}h_{m})⊙\text{sigm}(U_{a}h_{m}^{⊺}))\}}{\sum_{m=1}^{M}\text{exp}\{W_{a}(\text{tanh}(V_{a}h_{m})⊙\text{sigm}(U_{a}h_{m}^{⊺}))\}}$$

Once the attention scores for all slice images of the patient are obtained, the attention scores $a_{m}$ are utilized as weighting factors to modulate each slice image feature ${\text{h}}_{m}\in {\mathbb{R}}^{512}.\;$This results in the patient-level feature representation ${\text{h}}_{patient}\in {\mathbb{R}}^{512}$.


$$h_{patient}=\text{Attn pool}(A,H)=\sum_{m}^{M}a_{m}h_{m}$$

The patient-level feature representation consolidates key information from all the slices and is subsequently used for categorical prediction of the patient’s MSI versus MSS status (**Supplementary Figure S1**).

#### 2.3.2 Late fusion and intermediate fusion

The Late Fusion strategy, also known as Decision-Level Fusion, involves training a separate model for each modality and then combining the predictions from each submodule. In this approach, each sub-model independently performs feature extraction and prediction, and the final classification results are obtained by methods such as weighted averaging or other synthesis techniques (40) (**Supplementary Figure S2A**).

In contrast, in the intermediate fusion strategy, the feature extraction and fusion processes are highly coupled. The optimization of the learning process occurs throughout the entire model, during training, the model’s loss function not only influences the output layer of the classifier but also affects the feature extraction process through backpropagation. This enables the model to optimize feature extraction, making the extracted features more discriminative and representative in the fused representation, thereby improving overall classification performance (40) (**Supplementary Figure S2B**).

In this study, we adopted a fusion modeling approach to enhance the classification accuracy for predicting patients’ MSI versus MSS status. Specifically, we incorporated both late fusion and intermediate fusion strategies into the model design, where a detailed description of the intermediate fusion strategy is provided in Section 2.3.3.

#### 2.3.3 Progressive fusion

In exploring intermediate fusion strategies, this paper focuses on a deep learning-based multimodal fusion model (MMF) (**Supplementary Figure S3**). The model utilizes an attentional multi-instance learning (AMIL) framework to extract image feature vectors $h_{patient}$. Simultaneously, key features from gene expression data are extracted using differential gene analysis and transformed into molecular feature vectors $h_{omic}$ through a multilayer perceptron (MLP).

Prior to fusion, the image and molecular modalities are respectively subjected to a linear transformation and ReLU activation, resulting in the unimodal image feature $h_{histology}\in {\mathbb{R}}^{32}$ and molecular feature $h_{omic}\in {\mathbb{R}}^{32}$(see Equation 1). To fuse the features from both modalities, we add an element with a value of 1 to each unimodal feature vector. This step ensures the preservation of the original unimodal feature information during the fusion process. Subsequently, we apply the Kronecker product for deep fusion of the multimodal features, capturing the complex interaction between the two unimodal feature sets (41).

Specifically, the fusion matrix obtained by expanding the Kronecker product retains the original features of the molecular and image modalities in its last row and last column, respectively, while the remaining elements capture fine-grained interactions between the two modalities. This design enables effective preservation of unimodal information and significantly enhances the model’s ability to learn complex relationships across modalities.

(1) $$h_{fusion}=[\begin{array}{c} h_{histology}\\ 1 \end{array}]⊗[\begin{array}{c} h_{omic}\\ 1 \end{array}]=[\begin{array}{c} h_{1}\\ h_{2}\\ \vdots \\ h_{32}\\ 1 \end{array}]⊗[h_{1}^{'}\;h_{2}^{'}\;\cdots h_{32}^{'}\;1]=[\begin{array}{ccccc} h_{1}h_{1}^{'} & h_{1}h_{2}^{'} & \cdots & h_{1}h_{32}^{'} & h_{1}\\ h_{2}h_{1}^{'} & h_{2}h_{2}^{'} & \cdots & h_{2}h_{32}^{'} & h_{2}\\ \vdots & \vdots & \ddots & \vdots & \vdots \\ h_{1}^{'} & h_{2}^{'} & \cdots & h_{32}^{'} & 1 \end{array}]$$

**Figure 1:**
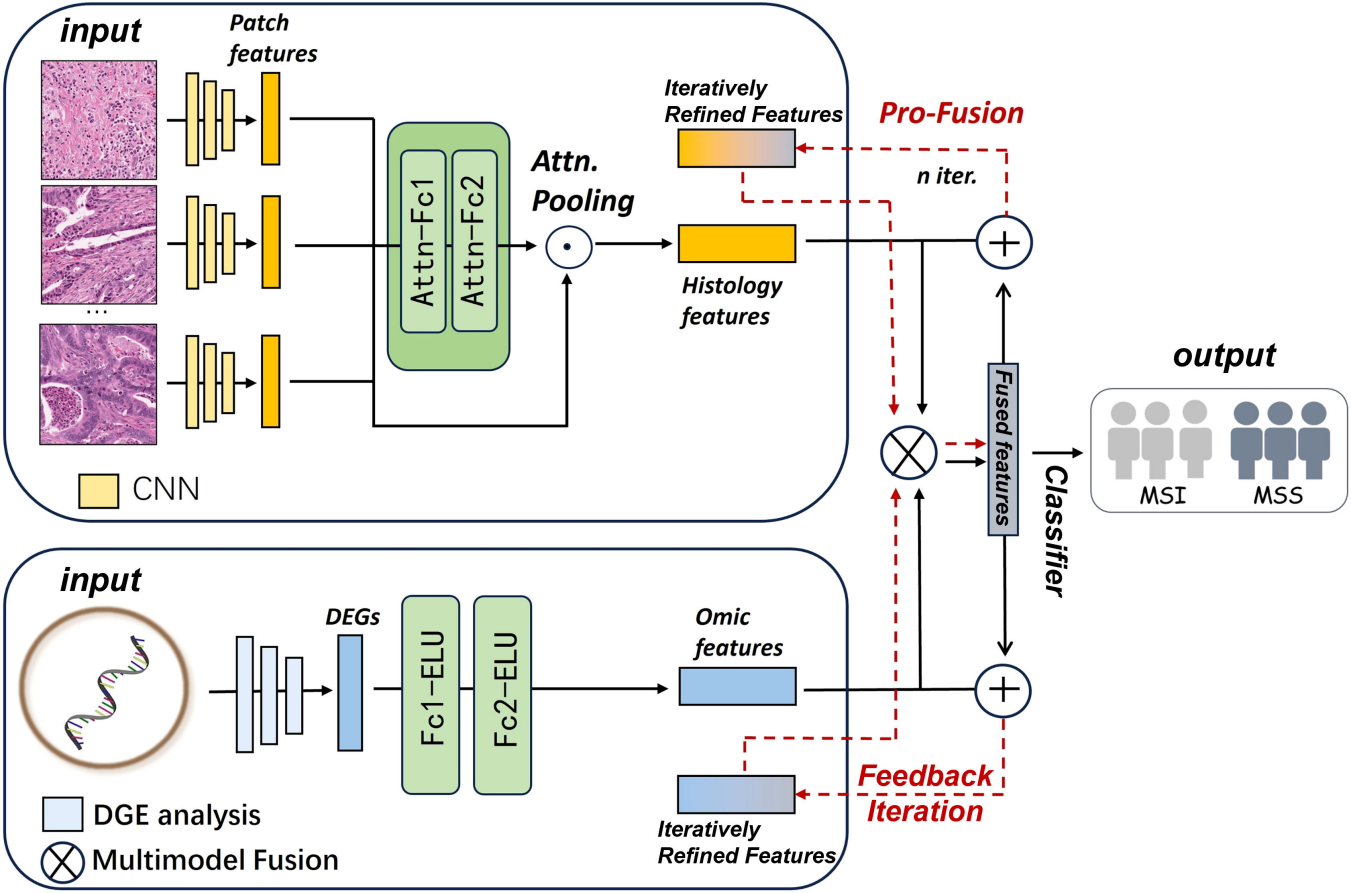
Multimodal data fusion (ProMMF). The ProMMF model adds a new back-projection operation based on the MMF model, which remaps the fused features back to the original image and genome space and sums them with the original unimodal features to perform multimodal feature fusion again. This process is repeated for several iterations to enhance the fusion effect.

The reverse projection mechanism is a method of combining fused features with the original features to create an updated feature representation (42). To enhance the interaction and iterative refinement between multimodal features, this study introduces a projection mechanism into the fusion module of the existing MMF model, and proposes a novel progressive multimodal fusion algorithm (ProMMF) (**Figure 1**).

Specifically, in the ProMMF model, the reverse projection operation is illustrated by the red dashed lines in **Figure 1**. After the initial fusion of image modality features $h_{histology}$ and molecular modality features $h_{omic}$, the resulting joint representation is projected back into the original image and genomic feature spaces via two separate linear transformations, producing two reverse projection vectors, ${\hat{h}}_{histology}$ and ${\hat{h}}_{omic}$. This reverse projection can be viewed as a feature transformation mechanism, implemented through linear operations with learnable parameter matrices. The generated context vectors serve as cross-modal feedback, and are added to the original unimodal features before being re-fed into the fusion module, resulting in more expressive multimodal representations. This mechanism can be unrolled for multiple iterations to facilitate continuous alignment and enhancement of multimodal representations. The number of iterations was treated as a hyperparameter and searched over the range 1–5. The configuration with two iterations produced the highest AUC and F1 and was therefore adopted for all experiments. The detailed performance metrics across all iterations are shown in **Supplementary Figure S4**.

### 2.4 Paired bootstrap and permutation testing for AUC comparison

We employed a combined paired-bootstrap and permutation testing procedure to robustly compare the AUCs of two classification models. We performed resampling with replacement on the test set, applying identical sample indices to both models in each bootstrap replicate to preserve pairing and estimate the sampling distribution of the AUC difference. A total of 2,000 bootstrap replicates were generated, with 95% confidence intervals for each model’s AUC calculated using the percentile method (2.5th and 97.5th percentiles of the bootstrap distribution).

To assess the statistical significance of AUC differences, we conducted a paired permutation test with 5,000 replicates, where each observation’s model scores were independently swapped with probability 0.5 per permutation. The resulting permutation distribution of mean differences was used to compute two-sided p-values. Effect sizes were quantified using paired Cohen’s d, calculated as the mean pairwise difference divided by the sample standard deviation of those differences. Finally, statistical power was estimated through a parametric Monte Carlo procedure that simulated replicates from a normal distribution parameterized by the observed mean difference and standard deviation, reporting the proportion of simulated replicates exceeding the critical value at α = 0.05.

## 3 Results

### 3.1 General framework

We propose a systematic two-stage methodology. The first is the feature selection phase, where key genetic features are identified from the training set by differential gene analysis, while pathology image features are extracted using a pre-trained ResNet50 network. The second is the modeling phase, in which comparative experiments between unimodal and multimodal models were conducted. In the unimodal model, the modeling and classification prediction of pathology image data and gene expression data are performed separately, while in the multimodal model, the pathology image data and gene expression data are fused together and unified for classification prediction.

Specifically, unimodal models include the AMIL model based on pathology images and the DMLP model based on molecular data. The DMLP model uses differentially expressed genes (DEGs) identified through gene analysis as inputs and outputs classification predictions after a multilayer perceptron (MLP) (**Supplementary Table S3**).

Among the multimodal models, the late fusion model combines the prediction probabilities of the two unimodal models through weighted fusion to generate the final classification results. For the intermediate fusion MMF model, two fusion methods are employed: Kronecker and direct concatenation (Concat), labeled as MMF_Kron and MMF_Con, respectively. Similarly, the fusion strategy based on the ProMMF model also utilizes these two fusion methods, named ProMMF_Kron and ProMMF_Con (**Supplementary Table S3**). **Supplementary Table S3** also documents the dimensional configurations of the fusion layers and the corresponding dropout rates for each model.

### 3.2 Screening differentially expressed genes

**Figure 2:**
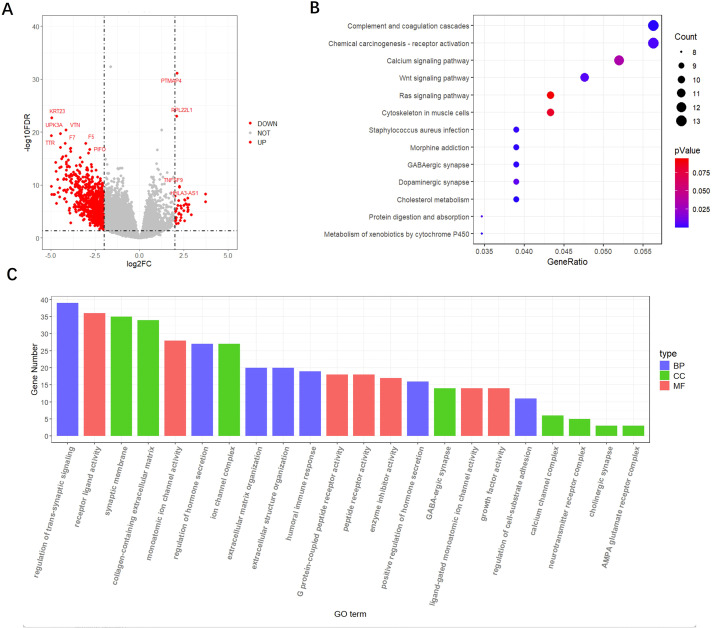
Differential gene analysis and enrichment analysis. **(A)** Volcano map of differentially expressed genes. The red dots represent differentially expressed genes which |logFC|>2 and p<0.05, and the gray dots represent genes that are not significantly or |logFC|<=2. **(B)** KEGG enrichment plot of differential genes. The size of the circle represents the number of genes enriched in the corresponding pathway. **(C)** GO enrichment plot of differential genes. The horizontal coordinate indicates the category of gene function and the vertical coordinate indicates the number of genes enriched to that function. The three colors represent three functional aspects: biological process (BP), cellular component (CC) and molecular function (MF).

After dividing the training set patients into MSI and MSS groups, we calculated the log Fold Change(logFC) and p-value of each gene between the two groups. We identified a total of 861 differentially expressed genes based on the thresholds of |logFC| > 2 and p < 0.05, including 41 up-regulated genes and 820 down-regulated genes, these genes were considered to exhibit significant differences in expression between the MSI and MSS groups. In the volcano plot (**Figure 2A**), the red dots represent the significantly differentially expressed genes, with partial gene name annotations. For example, PTMAP4, RPL22L1 and TNFSF9 are up-regulated genes, whereas KRT23, UPK3A and VTN are down-regulated genes.

### 3.3 KEGG and GO enrichment analysis

Through KEGG enrichment analysis of the screened differential genes, we identified their close association with several key immune signaling pathways (**Figure 2B**), including the Wnt signaling pathway, Ras signaling pathway, and the complement and coagulation cascade. These pathways play crucial roles in immunotherapy. The Wnt signaling pathway is pivotal in regulating the tumor microenvironment and immune escape. Aberrant Wnt signaling can lead to an immunosuppressive microenvironment, which impairs the efficacy of immunotherapy. The Ras signaling pathway influences immunotherapy efficacy by promoting the proliferation and survival of tumor cells, and its activation may diminish the immune system’s response. The complement and coagulation cascades are integral components of the innate immune system, regulating immune responses. Complement activation can either enhance the anti-tumor immune response or contribute to immune escape, thereby influencing the effectiveness of immunotherapy.

Meanwhile, the results of GO analysis further revealed the roles of these differential genes in the immune response, which were mainly involved in three aspects, namely, biological process (BP), cellular component (CC) and molecular function (MF) (**Figure 2C**). First, regarding BP, processes such as humoral immune response, regulation of extracellular matrix organization, and regulation of cell-substrate adhesion directly influence immune cell activation, infiltration, and immune escape mechanisms in the tumor microenvironment. For example, the humoral immune response enhances T-cell activity through antibody production, whereas changes in the extracellular matrix determine the infiltration capacity of immune cells, an important factor influencing immunotherapy. At the CC level, components such as synaptic membranes and collagen-containing extracellular matrix are involved in regulating immune cell migration and immune responses in the tumor microenvironment, affecting the efficacy of immunotherapy. At the MF level, such as receptor-ligand activity, G protein-coupled peptide receptor activity, and monoatomic ion channel activity, receptor-ligand interactions blocked the tumor immune escape mechanism in the treatment of immune checkpoint inhibitors, whereas modulation of ion channel function enhanced immune cell anti-tumor activity.

### 3.4 Training and testing models for MSI versus MSS status prediction

The ProMMF_Kron model consists of an AMIL network, an MLP network, and a multimodal fusion layer, which consists of two aggregation methods, Kronecker, Concat, and a back projection module. Dropout regularization with a rate of 0.1 was applied to critical network components to mitigate overfitting. During the training process, Adam was used as the optimizer with a learning rate of $1\times 10^{-4}$ and a weight decay of $5\times 10^{-3}$ for 100 epochs, with binary cross entropy loss employed as the optimization objective. The batch size was set to 1 due to the different number of images in each sample, the image features and molecular features of the same patient were randomly extracted each time.

**Figure 3:**
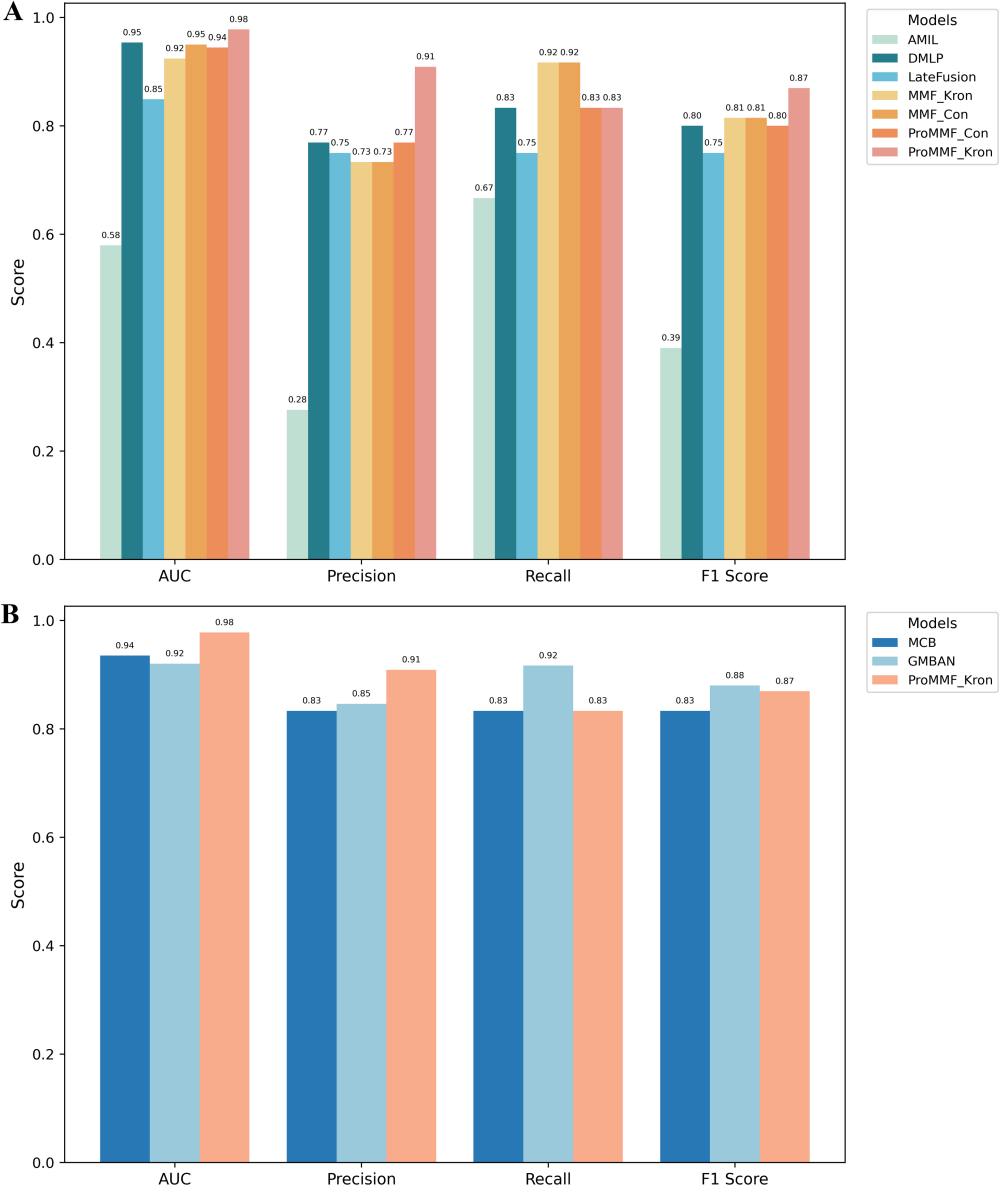
Performance comparison of different models on AUC, Precision, Recall, and F1-score metrics. **(A)** shows the results of unimodal (AMIL, DMLP) and multimodal (LateFusion, MMF_Kron, MMF_Con, ProMMF_Con, ProMMF_Kron) models. **(B)** compares our ProMMF_Kron with MCB and GMBNA multimodal models.

The performance of unimodal and multimodal models in predicting the MSI versus MSS status of patients was evaluated on the test set, and the results showed that the performance of different models in the task of MSI versus MSS classification of STAD patients varied widely (**Figure 3A**; **Supplementary Table S4**).

The AMIL model, which relies solely on image data, performed poorly overall, with an AUC of only 0.58, indicating its weak ability to distinguish between sample categories. In contrast, the DMLP model, based on gene expression data, outperformed the AMIL model across all evaluation metrics, with a significant increase in AUC to 0.95, demonstrating the effectiveness of gene expression data for this task.

Multimodal fusion models generally outperformed unimodal models, with the ProMMF_Kron model achieving the best performance across all key metrics, showcasing its superior predictive capability. Specifically, ProMMF_Kron attained the highest AUC of 0.98, indicating its excellent ability to distinguish between positive and negative samples. Additionally, its Precision of 0.91 suggests a high accuracy in predicting positive samples, with a low false positive rate. Furthermore, ProMMF_Kron’s F1 score of 0.87 reflects a well-balanced trade-off between Precision and Recall. Notably, the MMF_Kron and MMF_Con models both achieved the highest Recall of 0.92. However, they underperformed ProMMF_Kron in other critical metrics; specifically, our model demonstrated an improvement of 0.06 in both AUC and F1 score over the standard Kronecker fusion (MMF_Kron). This resulted in an overall inferior performance compared to ProMMF_Kron. In contrast, the late fusion model exhibited relatively poor performance, with an AUC of only 0.85, which was even lower than the unimodal gene expression model DMLP(AUC = 0.95). This may be attributed to late fusion’s failure to fully exploit the complementary information between different modalities, leading to its inability to enhance overall predictive performance effectively. To further assess the robustness of model performance, we computed the 95% confidence intervals (CIs) for the AUC metrics (see **Supplementary Table S4**). The ProMMF_Kron model demonstrated the best overall performance, a conclusion that is strongly supported by its superior AUC point estimate and its precise and high-lying confidence interval. In conclusion, the ProMMF_Kron model demonstrated the best overall performance in this study, validating the effectiveness of the proposed Pro-Fusion strategy.

We evaluated stage-specific performance by stratifying the test set according to AJCC pathologic stage into early (I–II) and late (III–IV) groups, and applied the trained ProMMF_Kron model to each subgroup using the same testing protocol. Results are summarized in **Supplementary Table S5**: for early-stage samples the model yielded AUC = 0.96 and F1 = 0.89; for late-stage samples it yielded AUC = 1.00 and F1 = 0.75. To further examine potential confounding factors such as previous therapeutic interventions, additional subgroup analyses were performed. For previously treated patients, the model achieved AUC = 1.00 and F1 = 0.80; for treatment-naïve patients, it achieved AUC = 0.97 and F1 = 0.75 (**Supplementary Table S6**). These findings indicate robust discriminative performance across both stage and treatment subgroups.

To validate the stability and generalization capability of the ProMMF_Kron model, we conducted five-fold cross-validation. This approach evaluates the model's average performance across different data partitions, effectively mitigating bias caused by randomness when the sample size is relatively limited. The results demonstrated that the ProMMF_Kron model exhibited low variance across all metrics: mean AUC of 0.9572±0.0243, Precision of 0.8812±0.0506, Recall of 0.9052±0.0634, and F1-score of 0.8905±0.0201. These findings indicate consistent model performance across different data subsets, demonstrating strong robustness and generalization capability (**Supplementary Table S7**).

To assess the model’s cross-cancer applicability, we applied the same model framework used for TCGA-STAD to colorectal cancer (CRC) by retraining it on CRC data. Using 357 CRC cases with matched histopathology and gene expression data, we performed rigorous validation with patient-level stratified splits (6:2:2 ratio, random_state = 2024). Notably, the model achieved outstanding performance on the independent test set, with an AUC of 0.99, Precision of 1.00, Recall of 0.92, and F1-score of 0.96. This performance was comparable to that in the original gastric cancer setting, confirming not only the stability of the method but also its practical value for pan-cancer feature extraction.

### 3.5 Comparative analysis with existing multimodal models

To evaluate the effectiveness of ProMMF_Kron, we compared its performance against two state-of-the-art multimodal models: the Gated Multimodal Bi-Attention Network (GMBAN) proposed by Kayikci et al. (44) and Multimodal Compact Bilinear pooling method (MCB) introduced by Qiu et al. (36), using identical training and testing sets (**Figure 3B**; **Supplementary Table S8**). ProMMF_Kron exhibited competitive performance, particularly in AUC and precision.

Specifically, ProMMF_Kron achieved the highest AUC (0.98), significantly outperforming GMBAN (0.94) and MCB (0.92), demonstrating its enhanced discriminative capability in classifying MSI *vs*. MSS samples. While MCB attained the best recall (0.92), ProMMF_Kron maintained a high precision (0.91) with a balanced recall (0.83), yielding an F1 score (0.87) comparable to MCB (0.88) and surpassing GMBAN (0.83).

To rigorously quantify the superiority of our proposed ProMMF_Kron framework, we performed a statistical comparison against all three baseline models, including a special emphasis on DMLP as it represented the strongest-performing unimodal benchmark. As summarized in **Supplementary Table S9**, ProMMF_Kron achieved a statistically significant improvement in AUC (p < 0.001 for all comparisons) with large effect sizes (Cohen’s d > 0.8), notably outperforming even this best unimodal approach by a margin of 0.038. The statistical power for these comparisons reached 100%, confirming the high reliability of these findings.

In addition to predictive performance, we evaluated the computational efficiency of each model. As detailed in **Supplementary Table S10**, ProMMF_Kron demonstrated remarkable parameter efficiency, containing only 4.5 million parameters. This represents a 4.7-fold and 2.8-fold reduction in model size compared to MCB (21.4M params) and GMBAN (12.5M params), respectively. In terms of training time, ProMMF_Kron was faster than MCB, though it was slower than GMBAN model, which achieved the fastest training time.

These results underscore the overall advantages of ProMMF_Kron, validating the efficacy of its Pro-Fusion mechanism in delivering superior predictive performance for MSI versus MSS classification while maintaining high computational efficiency. ProMMF_Kron achieves an optimal balance between accuracy and complexity for potential clinical applications.

### 3.6 SHAP feature visualization and correlation analysis

**Figure 4:**
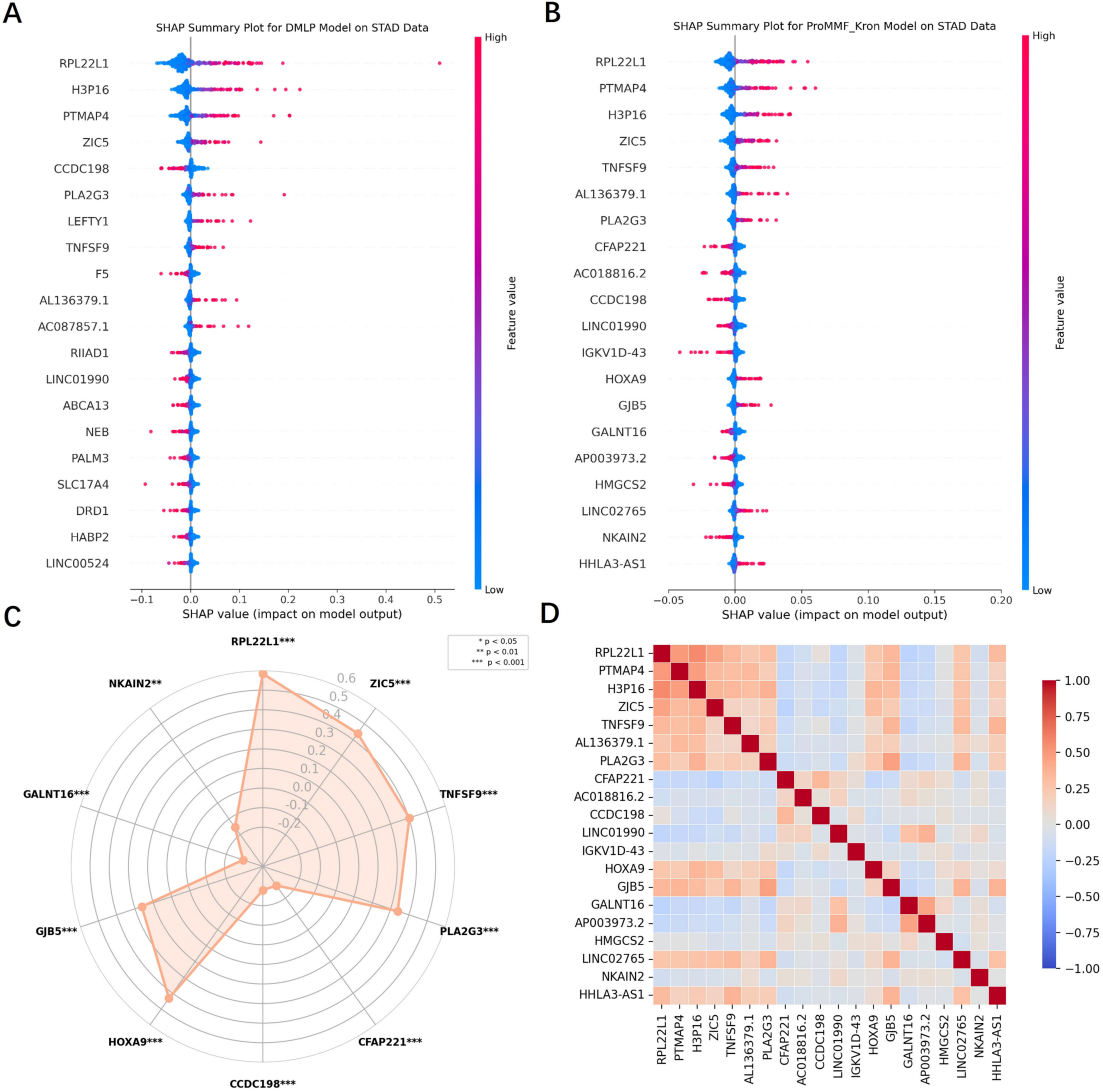
SHAP Gene visualization and correlation analysis. **(A)** Visualization of significant genes in the unimodal model DMLP. **(B)** Visualization of significant genes in the multimodal model ProMMF_Kron. Each dot represents a patient, and the red color indicates that the patient has high expression on the gene. When the red dot is on the right side, it indicates that the high expression of the gene is more likely to predict that the patient has MSI status; when the red dot is on the left side, it indicates that the high expression of the gene is more likely to predict that the patient has MSS status. **(C)** The SHAP plot demonstrates some of the important genes in the model predictions and shows the correlation of these genes with MSI through a radar plot. The circle values on the radar plot indicate the specific correlation magnitude. **(D)** Correlation heatmap of significant genes, where darker red color indicates stronger positive correlation and darker blue color indicates stronger negative correlation.

In this study, we also visualized the important gene features for both unimodal and multimodal models. Notable differences were observed in the key features identified by the two models. Compared to the SHAP plot of the DMLP model (**Figure 4A**), the ProMMF_Kron showed a more significant impact on identifying key gene features after incorporating image data. The SHAP plot for the ProMMF_Kron revealed that genes such as RPL22L1, PTMAP4, and H3P16, with high expression levels, were more likely to be predicted as MSI, while genes with high expression, such as CFAP221 and CCDC198, were more likely to be predicted as MSS (**Figure 4B**).

To further validate these findings, we analyzed the correlation between key genes and patients’ MSI versus MSS status using an online tool developed by Liao et al. (43) and generated a radar plot (**Figure 4C**). The results showed that the RPL22L1 gene was positively correlated with MSI status, consistent with our model’s prediction, where patients with high expression of RPL22L1 were classified as MSI. The correlation results for other genes were similar, further validating the accuracy of our SHAP analysis.

In this study, we also generated a correlation heatmap for the important genes mentioned above (**Figure 4D**). The results showed that most of the genes have weak correlations with each other. This weak correlation may indicate that the relationships between these genes are not simply linear but rather more complex and nonlinear. Since our model can capture nonlinear features, it can still utilize the important information from these genes for prediction, even though their linear correlations are weak. Notably, these genes have large weights in the model but low correlations with each other, suggesting that they may represent independent novel biomarkers that can individually influence the prediction outcomes and are less likely to be affected by the expression levels of other genes.

## 4 Discussion

MSI has been widely recognized as a prognostic indicator for immunotherapy response in STAD patients. However, due to the complexity of its detection method, not every patient can undergo MSI testing. Therefore, utilizing machine learning models for assisted prediction becomes particularly important.

Compared with unimodal feature models, our proposed ProMMF_Kron integrates gene sequencing data (providing molecular-level insights) and histopathological images (revealing tissue morphology), leveraging their complementary strengths for more comprehensive tumor characterization. This multimodal approach underscores the critical role of combining diverse data sources in predicting immunotherapy response.

The Pro-Fusion mechanism in ProMMF_Kron provides three major advantages (1). Adaptability — if fusion proves ineffective, the parameters can be zeroed during training, ensuring performance remains at least comparable to baseline models (2). Iterative refinement — multiple optimization rounds reinforce discriminative features while suppressing noise (3). Interpretability — structured cross-modal interactions offer clearer biological insights than implicit fusion methods.

To clarify why ProMMF_Kron achieves these improvements, we next analyze the theoretical basis of its Pro-Fusion mechanism. Pro-Fusion overcomes the limitations of early and late fusion through a Pro-Fusion mechanism: early fusion can preserve fine-grained cross-modal correlations but struggles with data heterogeneity, whereas late fusion alleviates heterogeneity issues at the cost of information loss. Our framework constructs a cross-modal iterative optimization loop, in which high-level fused features are fed back into unimodal encoders to enable conditional feature extraction. In this way, it retains the detailed interaction characteristics of early fusion while maintaining the modality adaptability of late fusion. Through multiple iterations, it progressively refines feature representations, ultimately achieving deep collaboration and precise interpretation of multimodal information.

When benchmarked against existing multimodal models, ProMMF_Kron achieves competitive performance. While GMBAN (44) relies on attention-based fusion and MCB (36) employs compact bilinear pooling, our model’s explicit interaction modeling and Pro-Fusion mechanism yield higher discriminative power (AUC: 0.98 *vs*. 0.94/0.92) while maintaining balanced precision-recall trade-offs (F1: 0.87 *vs*. 0.83/0.88).

GO/KEGG enrichment analyses indicated that SHAP-identified genes were involved in immune-related pathways. For example, RPL22L1 is up-regulated in dMMR/MSI contexts and linked to immune-active phenotypes (45); TNFSF9 functions as an immune co-stimulatory ligand and correlates with T cell infiltration in MSI-STAD (46); while KRT23, UPK3A, and VTN are implicated in extracellular matrix remodeling and immune evasion, suggesting potential roles in neoantigen presentation or recognition (47). Taken together, these findings suggest that our results reflect not only generic immune signaling but also MSI-specific biology tied to MMR deficiency and neoantigen load. We acknowledge that SHAP analysis reflects association rather than causation, and functional validation will be needed in future studies.

This study has two main limitations. First, the stringent requirements for paired histopathology images and gene expression data result in a currently limited sample size (particularly for multimodal matched samples), which may affect model performance in specific subgroup analyses. We have implemented rigorous validation strategies including five-fold cross-validation and cross-cancer migration experiments (e.g., from gastric to colorectal cancer) to maximize result reliability, and future work will expand validation cohorts through multicenter collaboration. Second, histopathology imaging relies on invasive sampling, which may cause patient discomfort and pose clinical challenges. Subsequent research will explore non-invasive alternatives (e.g., CT radiomics or liquid biopsies) and extend validation to additional cancer types. In addition, although our model showed promising technical performance, its clinical utility requires validation in prospective cohorts and real-world settings, which we acknowledge as a limitation. Since MSI can already be assessed by PCR/IHC, our approach is intended as a complementary computational tool whose value should be further evaluated with cost-effectiveness and feasibility analyses. Nevertheless, ProMMF_Kron establishes a robust framework for immunotherapy prediction by combining interpretable multimodal fusion with biological plausibility. This work not only provides methodological references for overcoming sample size limitations but also highlights the translational value of AI tools as complementary to traditional MSI testing in clinical decision-making.

## 5 Conclusion

In summary, this study integrates molecular biological information and pathological image data to perform classification prediction of MSI status in STAD patients. We propose a multimodal fusion model named ProMMF_Kron, which demonstrates competitive performance compared to various unimodal approaches. Notably, the introduction of the Pro-Fusion mechanism significantly enhances the fusion process, enabling ProMMF_Kron to outperform fusion models without this mechanism. Moreover, it achieves better results than existing state-of-the-art multimodal methods across key evaluation metrics such as AUC and precision, strongly validating the effectiveness and innovation of the proposed fusion strategy.

## Data Availability

The original contributions presented in the study are included in the article/**Supplementary Material**. Further inquiries can be directed to the corresponding author.
