# Supplementary material for: ProMMF_Kron: a multimodal deep learning model for immunotherapy response prediction in stomach adenocarcinoma
Source: Front Immunol. 2026 Feb 10;17:1602846. doi: 10.3389/fimmu.2026.1602846 (PMC12929529; doi:10.3389/fimmu.2026.1602846)
Supplement: Supplementary file 1 [file Supplementaryfile1.pdf]

Supplementary Material

1 Supplementary Figures and Tables

1.1 Supplementary Figures

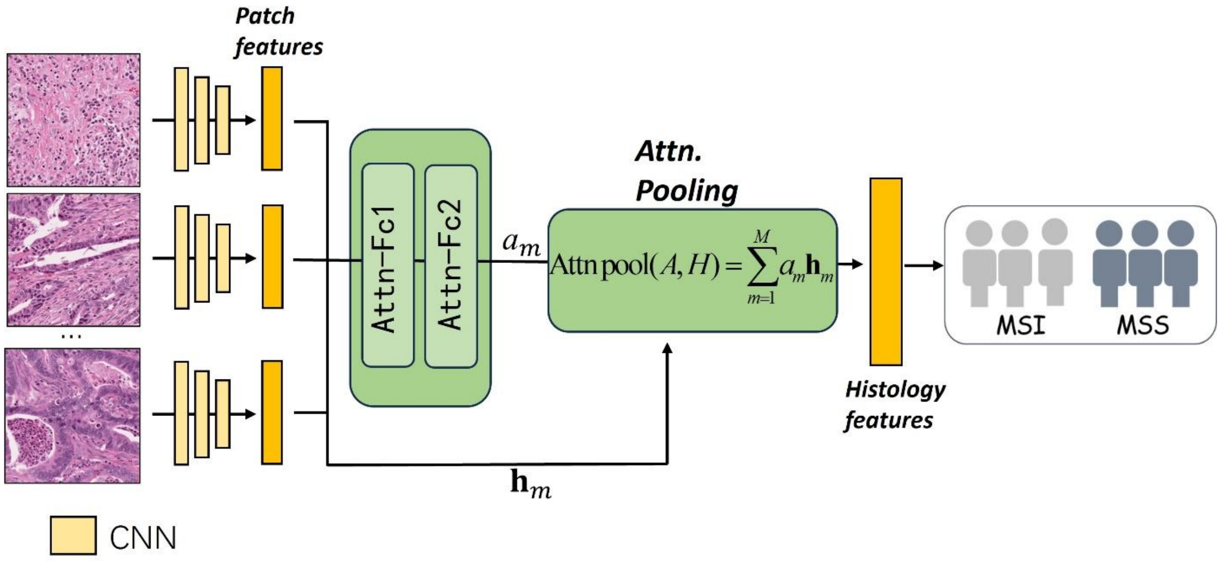

Figure S1. The pipeline of Attention-based deep multi-instance learning (AMIL). Convolutionally extract the features of all the blocks of each patient to get the vector representation of the block features, and then pool the features of all the blocks of each patient through attention pooling to get the patient-level feature representation.

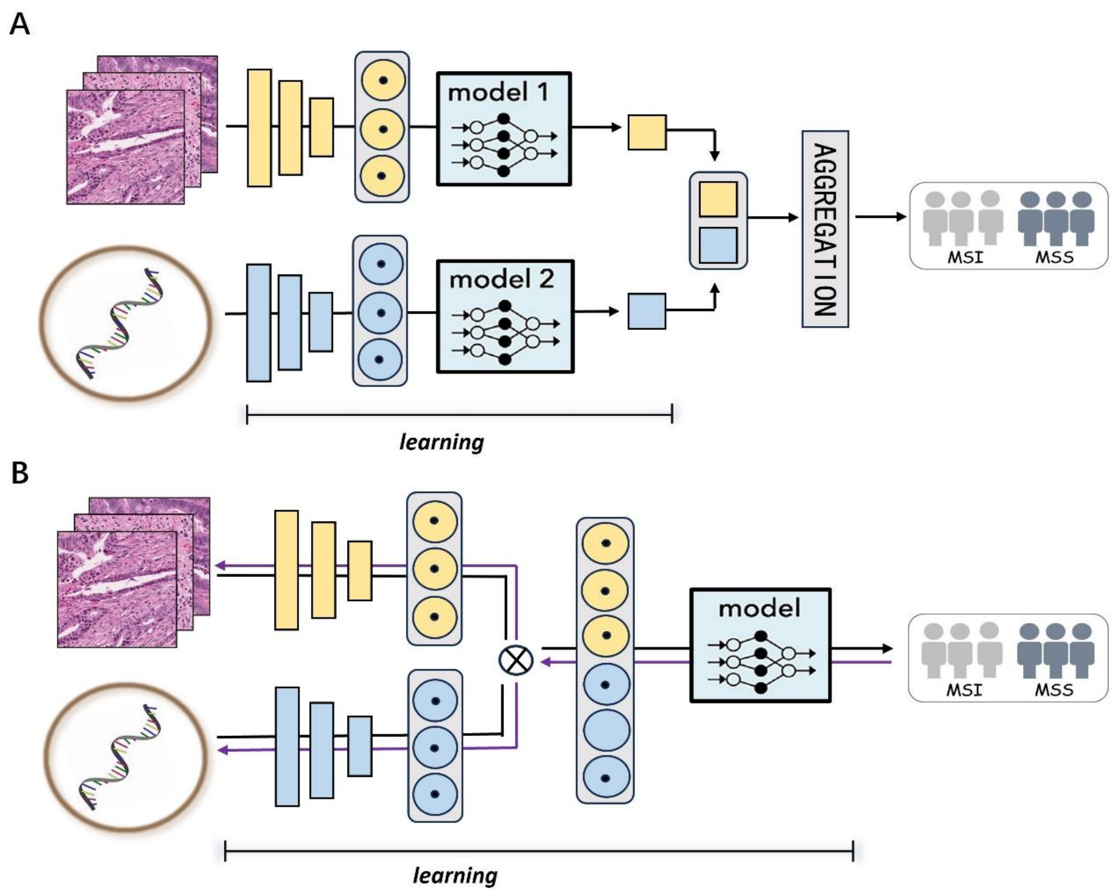

Figure S2. Late fusion and intermediate fusion: (A) Late fusion trains a separate model for each modality and aggregates the predictions of each model at the decision level. (B) Intermediate fusion losses are propagated to the feature extraction layer for each modality to optimize the feature extraction process.

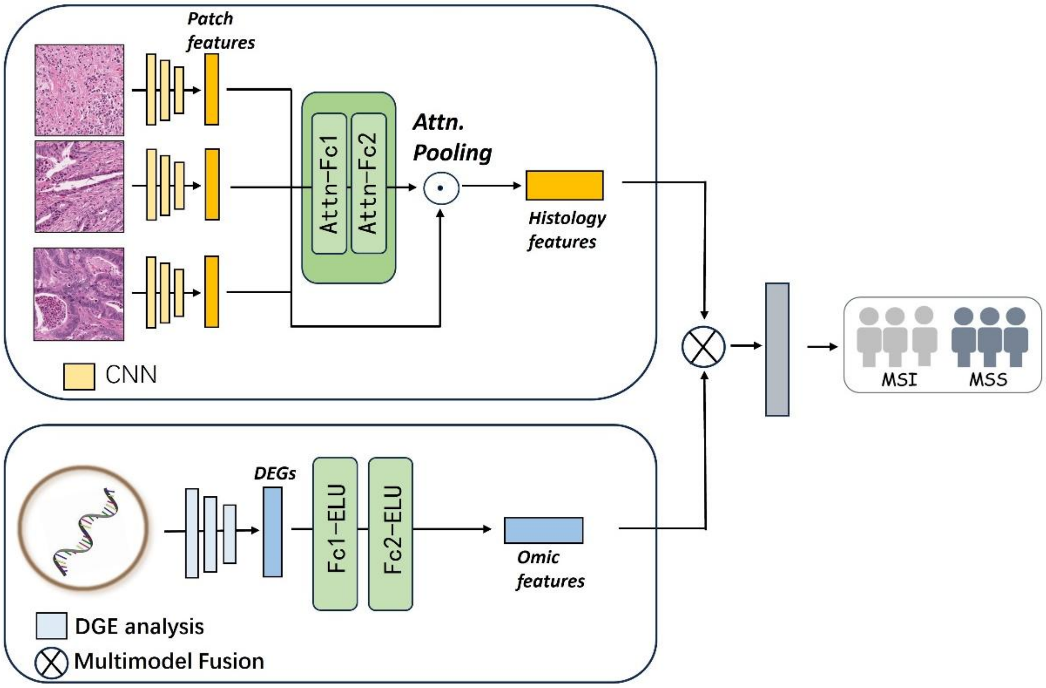

Figure S3. Multimodal data fusion (MMF). The MMF model consists of three parts, in which the AMIL network is used to aggregate the image data features, while the DMLP network is used to extract the gene expression data features, and finally the fusion of the two modal data is realized by the Kronecker product.

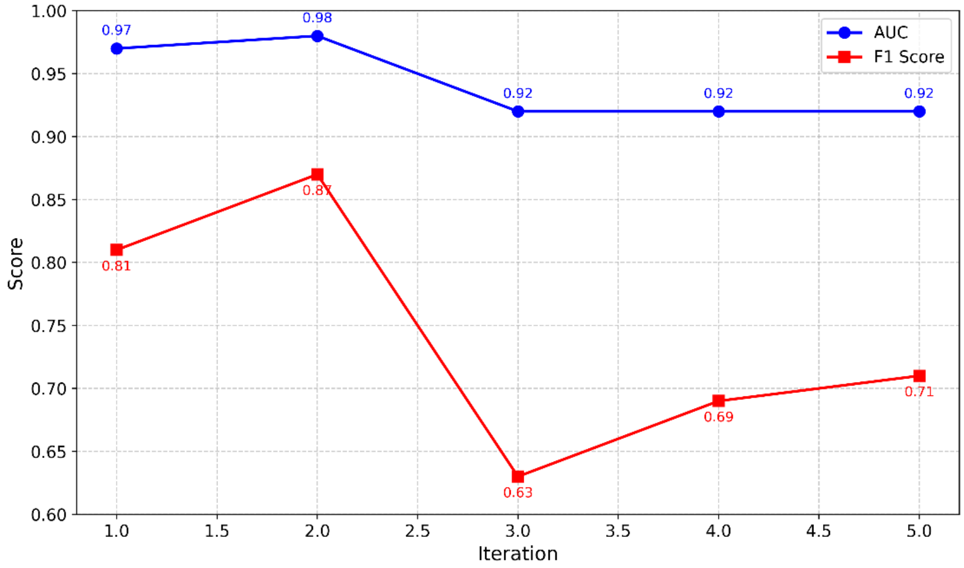

Figure S4. Performance metrics of ProMMF_Kron across multiple iterations. It illustrates the performance of ProMMF_Kron across different iteration configurations (1–5). The AUC (blue) and F1 score (red) are plotted for each iteration. ProMMF_Kron is Kronecker fusion based on ProMMF model.

1.2 Supplementary Tables

Table S1. Clinico-pathological variables of all patient cohorts.

	MSI	MSS
Subjects(n)	59	223
Age (year)	70.0 (9.1)	64.2 (10.7)
Gender		
Male	31(52.5%)	160(71.7%)
Female	28(47.5%)	63(28.3%)
Race		
White	34(57.6%)	143(64.1%)
Asian	12(20.3%)	51(22.9%)
Not reported	7(11.9%)	13(5.8%)
Black or african american	2(3.4%)	10(4.5%)
Unknown	4(6.8%)	6(2.7%)
Stage of disease		
Stage I	11(19.0%)	24(10.8%)
Stage II	20(34.4%)	54(24.2%)
Stage III	18(31.0%)	88(39.5%)
Stage IV	4(6.8%)	19(8.5%)
--	6(10.2%)	38(17.0%)
Previous therapeutic		
Yes	6(10.2%)	30(13.5%)
No	53(89.8%)	189(84.8%)
--	0(0.0%)	4 (1.8%)

Table S3. Comparison of Unimodal and Multimodal Architectures.

Modle	Data Type	Integration Strategies	Fusion Stage	Fusion hidden dim	Dropout Rate
DMLP	molecular data	-	-	-	0.25
AMIL	Image data	-	-	-	0.1
LateFusion	molecular and Image data	direct concatenation	Late Fusion	256	0.2
MMF_Kron	molecular and Image data	Kronecker	intermediate fusion	256	0.2
MMF_Con	molecular and Image data	direct concatenation	intermediate fusion	256	0.2
ProMMF_Kron	molecular and Image data	Kronecker	intermediate fusion	256	0.2
ProMMF_Con	molecular and Image data	direct concatenation	intermediate fusion	256	0.2

AMIL: Unimodal model based on image data; DMLP: Unimodal model based on gene expression data; MMF_Kron: Kronecker fusion based on MMF model; LateFusion: Late fusion; MMF_Con: Direct concatenation fusion based on MMF model; ProMMF_Con: ProMMF model based on direct concatenation fusion; ProMMF_Kron: Kronecker fusion based on ProMMF model.

Table S4. Performance of each model on AUC, Precision, Recall and F1_score metrics.

Model	AUC (95% Confidence Interval)	Precision	Recall	F1 Score
AMIL	0.58 ([0.38,0.79])	0.27	0.67	0.39
DMLP	0.93 ([0.80,1.00])	0.69	**0.92**	0.79
LateFusion	0.85 ([0.71,1.00])	0.75	0.75	0.75
MMF_Kron	0.92 ([0.75,1.00])	0.73	**0.92**	0.81
MMF_Con	0.95 ([0.74,1.00])	0.73	**0.92**	0.81
ProMMF_Con	0.94 ([0.86,1.00])	0.77	0.83	0.80
ProMMF_Kron	**0.96 (**[0.89,1.00])	**0.91**	0.83	**0.87**

AMIL: Unimodal model based on image data; DMLP: Unimodal model based on gene expression data; MMF_Kron: Kronecker fusion based on MMF model; LateFusion: Late fusion; MMF_Con: Direct concatenation fusion based on MMF model; ProMMF_Con: ProMMF model based on direct concatenation fusion; ProMMF_Kron: Kronecker fusion based on ProMMF model.

Table S5. Performance stratified by clinical stage (STAD).

Clinical stage	AUC	Precision	Recall	F1 Score
Early (I–II)	0.96	1.00	0.80	0.89
Late (III–IV)	1.00	0.60	1.00	0.75

Table S6. Performance stratified by prior therapeutic intervention (STAD).

Previous treatment history	AUC	Precision	Recall	F1 Score
Yes	1.00	1.00	0.67	0.80
Noterial	0.97	0.75	0.75	0.75

Table S7. Performance Metrics Across 5-Fold Cross-Validation.

Fold	Fold 1	Fold 2	Fold 3	Fold 4	Fold 5	Mean	Std
AUC	0.9787	0.9626	0.9205	0.9782	0.9458	0.9572	0.0245
Precision	0.8182	0.9286	0.9091	0.9167	0.8333	0.8812	0.0514
Recall	1	0.8667	0.8333	0.9167	0.9091	0.9052	0.0628
F1 Score	0.9	0.8966	0.8696	0.9167	0.8696	0.8905	0.0205

Table S8. Performance of GMBAN, MCB, and ProMMF_Kron.

Model	AUC	Precision	Recall	F1 Score
GMBAN	0.94	0.83	0.83	0.83
MCB	0.92	0.85	**0.92**	**0.88**
ProMMF_Kron	**0.98**	**0.91**	0.83	0.87

Table S9. Statistical Comparison of ProMMF_Kron against Baseline Models.

Comparison Model	Mean AUC (ProMMF_Kron)	Mean AUC (Baseline)	Mean Difference (ΔAUC)	95% CI of ΔAUC	p-value	Effect Size (Cohen's d)	Statistical Power (1-β)
MCB	0.964 ± 0.03	0.918 ± 0.074	0.046	[-0.007, 0.164]	<0.001	0.99 (Large)	100%
GMBAN	0.964 ± 0.03	0.937 ± 0.059	0.027	[-0.015, 0.111]	<0.001	0.83 (Large)	100%
DMLP	0.964 ± 0.03	0.926 ± 0.056	0.038	[-0.008, 0.115]	<0.001	1.22 (Large)	100%

Notes: AUC, area under the curve; CI, confidence interval. p-values were derived from a paired permutation test with 5000 iterations. Effect size was interpreted as per Cohen's guidelines: $|d| \geq 0.8$ indicates a large effect.

Table S10. Model Complexity and Training Efficiency Comparison.

Model	Total Parameters	Training Time (100 Epochs)
GMBAN	12,492,994	05 min 37 sec
MCB	21,378,898	16 min 21 sec
ProMMF_Kron	4,503,202	10 min 08 sec
